# An updated analysis of the association between CD2-associated protein gene rs9349407 polymorphism and Alzheimer’s disease in Chinese population

**DOI:** 10.3389/fninf.2022.1006164

**Published:** 2022-10-20

**Authors:** Shan Gao, Jia-wei Hao, Ya-nan Zhao, Xuan Li, Tao Wang, Zhi-fa Han, Bao-liang Sun, Jing-yi Sun, Gui-you Liu

**Affiliations:** ^1^Beijing Institute of Brain Disorders, Laboratory of Brain Disorders, Ministry of Science and Technology, Collaborative Innovation Center for Brain Disorders, Capital Medical University, Beijing, China; ^2^Department of Interventional Radiology and Vascular Surgery, Affiliated Hospital of Hebei University, Baoding, Hebei, China; ^3^Academy for Advanced Interdisciplinary Studies, Peking University, Beijing, China; ^4^School of Medicine, School of Pharmaceutical Sciences, Tsinghua University-Peking University Center for Life Sciences, Tsinghua University, Beijing, China; ^5^Shandong Academy of Medical Sciences, The Second Affiliated Hospital, Shandong First Medical University, Taian, Shandong, China; ^6^Shandong Provincial Hospital Affiliated to Shandong First Medical University, Jinan, Shandong, China; ^7^Beijing Key Laboratory of Hypoxia Translational Medicine, National Engineering Laboratory of Internet Medical Diagnosis and Treatment Technology, Xuanwu Hospital, Capital Medical University, Beijing, China

**Keywords:** genome-wide association studies (GWAS), meta-analysis, Alzheimer’s disease (AD), CD2-associated protein (CD2AP), rs9349307 polymorphism, Chinese population

## Abstract

**Background:**

Since 2011, three large-scale genome-wide association studies (GWAS) have confirmed that the CD2AP rs9349407 polymorphism is significantly connected with Alzheimer’s disease (AD) in individuals of European descent. Subsequently, this association has been replicated in European populations, but is unclear whether it can be replicated in Chinese. Recently, the correlation between rs9349407 and AD in the Chinese population has become a research hotspot.

**Objective:**

To explore the association between rs9349407 polymorphism and AD in the Chinese population.

**Materials and methods:**

Firstly, based on the exclusion and inclusion criteria, we selected 11 independent studies from 8 articles exploring the correlation between rs9349407 variation and AD in Chinese. Secondly, we conducted a meta-analysis based on fixed and random effect models and conducted a heterogeneity test. Thirdly, we used the additive model, dominant model, and recessive model for subgroup analysis.

**Results:**

We demonstrated that the CD2AP rs9349407 polymorphism increases AD susceptibility in Chinese populations (OR = 1.33, 95% CI = 1.08–1.64, *P* = 7.45E-03), which is consistent with the effect observed in Caucasian populations. Additionally, subgroup analysis showed that rs9349407 under the additive model (GG + CC vs. GC, OR = 0.76, 95% CI = 0.61–0.97, *P* = 2.04E-02) and dominant model (GG + GC vs. CC, OR = 0.49, 95% CI = 0.32–0.74, *P* = 8.51E-04) were also significantly correlated with AD susceptibility, but not under the recessive model (GG vs. GC + CC, OR = 0.77, 95% CI = 0.58–1.03, *P* = 7.44E-02).

**Conclusion:**

These existing data suggest that rs9349307 is significantly correlated with the susceptibility to AD in the Chinese population, but future studies with large samples are needed to confirm our findings.

## Introduction

Alzheimer’s disease (AD) is a neurological illness with an insidious onset and progressive development (Lambert et al., 2013). It is characterized clinically by irreversible memory loss, cognitive decline, increasing memory impairment, and personality and behavioral abnormalities (Cummins et al., 2018). The pathological alterations are accompanied by Aβ-amyloid protein aggregation to generate senile plaques (SP) and aberrant phosphorylated microtubule-associated protein (Tau) aggregation to produce neurofibrillary tangles (NFTs) (Zhao et al., 2022). The incidence rate of AD was positively correlated with age. At the age of 65, AD is categorized into two categories: early-onset AD (EOAD) and late-onset AD (LOAD), with LOAD comprising roughly 94% of the total (Scheltens et al., 2021). AD affects roughly 10% of people over the age of 65 and 50% of those over the age of 85. According to figures, there were 52 million patients with AD worldwide in 2019, and China recorded the highest number at roughly 10 million, with the number anticipated to rise to 20 million by 2050 (Chan et al., 2013; Li and Otani, 2018). However, there is no specific and appropriate therapy for AD (Jiang et al., 2012; Xu et al., 2015; Li et al., 2016).

Since AD was first diagnosed in the early twentieth century, genetic risk factors and their roles in the pathogenesis of AD have become the focus of genetic association studies (Lambert et al., 2013). In addition to APOE, several large-scale genome-wide association studies (GWAS) conducted in European populations over the past decade have identified more than 30 common and rare novel AD susceptibility sites, including CD2AP (Bertram et al., 2008; Seshadri et al., 2010; Hollingworth et al., 2011; Lambert and Amouyel, 2011; Naj et al., 2011). These GWAS findings further promote candidate gene studies in other populations, especially in the Chinese population.

In a large-scale GWAS of AD, Hollingworth et al. (2011) originally discovered that CD2AP (rs9349407) was strongly correlated with the risk of LOAD in the Caucasian population (*P* = 1.20E-02). Another large 3-stage analysis of the GWAS datasets of European descent individuals by Naj et al. (2011) confirmed that rs9349407 in CD2AP contributes to increased LOAD risk (*P* = 1.20E-06). In contrast to the results of GWAS, Carrasquillo et al. (2011) identified no significant risk association between rs9349407 and AD susceptibility based on 2,634 individuals with AD and 4,201 healthy controls from 6 case-control populations of European ancestry (*P* = 7.20E-01). Simultaneously, numerous researchers have also extensively explored and researched the potential correlation between rs93349407 mutation of the CD2AP gene and genetic risk of AD in Asian populations.

Tan et al. (2013) in a case-control study containing the 612 LOAD and 612 controls, discovered that the replication results between CD2AP mutations and LOAD were inconsistent with the results of GWAS (*P* = 8.50E-01). In the following years, five independent studies by three scholars (Wang et al., 2016; Liu, 2020; Hou et al., 2021) found no significant correlation between CD2AP gene rs9349407 polymorphism and LOAD in the Han Chinese population and Mongolian population, respectively (Wang W: *P* = 3.38E-01; Wang E: *P* = 9.01E-02; Liu M: *P* = 2.24E-01; Liu H: *P* = 4.09E-01; Hou: *P* = 4.24E-01). It’s worth noting that Liu (2014) investigated 533 subjects in the Chinese population, with 215 with LOAD and 318 matched healthy controls. The results showed that rs9349407-C in CD2AP increased the risk of LOAD (*P* = 1.50E-02). Furthermore, three additional studies (Jiao et al., 2015; Xiao et al., 2015; Tao et al., 2017) in Chinese Han population provided overwhelming evidence to support the association of rs9349407 with LOAD risk (Jiao: *P* = 4.80E-02; Xiao 1: *P* = 8.38E-05; Xiao 2: *P* = 1.23E-04; Tao: *P* = 4.60E-11). In addition to the preceding study, the meta-analysis concluded by Chen et al. (2015) demonstrated that rs9349407 was a risk factor for LOAD (OR = 1.08, 95% CI = 1.05–1.12, *P* = 8.78E-07).

In conclusion, we found that previous and recent research on the association between the rs9349407 mutation of the CD2AP gene and the risk of AD in the Chinese population has not reached the same conclusion. The conflicting results may be influenced by several factors. The main reasons may be due to the lack of meaningful data caused by the small sample size of candidate gene research in each study and the genetic heterogeneity of the rs9349407 polymorphism in AD among different populations. Therefore, by collecting all published studies that meet the inclusion criteria in the Chinese population, we conducted a comprehensive and updated meta-analysis of 11 studies, including 4,173 AD individuals and 6,137 control individuals, to evaluate the relationship more accurately between the rs9349407 mutation of the CD2AP gene and the risk of AD in the Chinese population.

## Materials and methods

### Comprehensive literature search

We conducted a comprehensive literature search of five databases, including Google Scholar,^1^ China National Knowledge Infrastructure (CNKI^2^), PubMed,^3^ Web of science,^4^ and Wanfang.^5^ Keywords “AD,” “CD2AP,” “rs9349407,” and “China or Chinese” were used to select all possible studies. The literature search ended on 1 April 2022. In addition, we conducted a manual search of references cited in published articles to identify other studies that had been initially overlooked.

### Inclusion and exclusion criteria

The following criteria were used to determine the included published studies: (1) the selected studies were published before 1 April 2022; (2) the selected studies must evaluate the relationship between rs9349407 variation of the CD2AP gene and AD; (3) the experimental design of the included studies was case-control design and provided the number of cases in the case group and the control group; (4) the selected studies must provide the number of rs9349407 alleles or genotypes or sufficient data to calculate the number of rs9349407 genotypes, or (5) the included studies must provide an odds ratio (OR) and a 95% confidence interval (CI), or provide relevant data to calculate the OR and a 95% CI; Studies that do not meet either of the above inclusion criteria will be excluded.

### Data extraction

According to the inclusion criteria listed above, we independently and carefully extracted relevant information from all eligible studies. When there are different data extraction results, first try to solve them through discussion. If there is still no consensus, it is up to other authors to decide. Extracted data, including (1) the name of the first author; (2) the year of publication; (3) the population; (4) the numbers of AD cases and controls; (5) the numbers of rs9349407 genotype distribution of cases and controls; (6) basic information of the study, such as OR, 95% CI and *P*-value (if not provided, calculate OR and 95% CI).

### Genetic model

The rs9349407 polymorphism of the CD2AP gene contains two types of variants (G and C). G is the major allele and C is the minor allele. We hypothesized that G and C were high and low risk factors for AD, respectively. In this meta-analysis, we used an allele model to evaluate the relationship between rs9349407 and AD. Dominant model (GG + GC vs. CC), additive model (GG + CC vs. GC) and recessive model (GG vs. GC + CC) were also used for analysis of this study only in the studies that provided GG, GC, and CC genotype data (Lewis and Knight, 2012).

### Heterogeneity test

The heterogeneity between studies was assessed by Cochran’s *Q* test and *I*^2^ statistics {*I*^2^ = [Q-(K-1)]/Q × 100%}. The Q statistic approximately follows an *X*^2^ distribution with k-1 degrees of freedom (K is devoted to determining the number of studies available for analysis). *P* < 0.10 and *I*^2^ > 50% indicated the existence of heterogeneity. In addition, based on the results of the heterogeneity test, fixed-effect models or random-effect models were selected to calculate the OR and 95% CI of the meta-analysis to assess the degree of genetic association. If the *P*-value of the *Q* test is <0.10 and *I*^2^ > 50%, indicating significant heterogeneity, the random-effects model is used to calculate the OR of the meta-analysis. In contrast, if the heterogeneity between studies is not significant, a fixed-effects model is used. The significance of pooled OR was determined by the *Z* test, in which *P*_*Z*_ < 0.05 shows an obvious statistical difference. All statistical methods were performed using R.^6^

### Sensitivity and publication bias analyses

We determined the effect on the heterogeneity test by omitting each study in turn and performed sensitivity analyses to assess the stability of the overall results. Publication bias was weighted by funnel plot and Egger’s regression analysis (*P* < 0.05 represents significant publication bias) (Egger et al., 1997). In this study, R (see text footnote 6) was used for data analysis and processing.

## Results

### Comprehensive literature search

**FIGURE 1:**
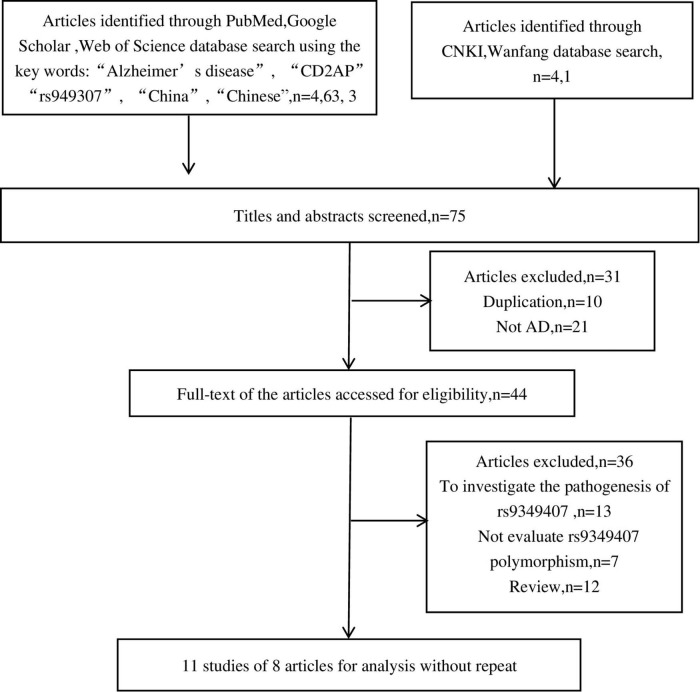
Flowchart of studies excluded or included in this meta-analysis.

As shown in Figure 1, we retrieved 4, 63, 3, 4, and 1 article from PubMed, Google Scholar, Web of Science, CNKI, and Wanfang databases, respectively. After a preliminary review of titles or abstracts, 31 articles were deleted due to duplication or irrelevant to this meta-analysis. Further reading of the full text revealed that 13 articles investigated the pathogenesis of rs9349407 and AD, 7 articles did not mention the rs9349407 mutation of the CD2AP gene, and 12 articles were related to meta-analyses and reviews. Ultimately, this meta-analysis included 11 studies from 8 articles as of April 2022, including 4,173 cases and 6,137 controls. The main features of the included studies are shown in Table 1.

**TABLE 1:** Characteristics of the correlation between the rs9349407 mutation of the CD2AP gene and AD in the Chinese population.

Study	Population	Case	Control	Case genotypes			Control genotypes		
					
				GG	GC	CC	GG	GC	CC
Tan et al., 2013	Northern	612	612	474	130	8	478	125	9
Liu, 2014	South China	239	207	NA	NA	NA	NA	NA	NA
Jiao et al., 2015	South China	229	318	NA	NA	NA	NA	NA	NA
Xiao et al., 2015	South China	459	751	NA	NA	NA	NA	NA	NA
Xiao et al., 2015	South China	459	751	NA	NA	NA	NA	NA	NA
Wang et al., 2016	East China	474	591	242	85	6	259	69	6
Wang et al., 2016	Southwest	533	1802	312	94	9	336	87	3
Tao et al., 2017	South China	393	383	267	115	11	110	214	14
Liu, 2020	China-Mong	506	385	398	94	14	307	77	1
Liu, 2020	China-Han	239	290	183	49	7	148	41	1
Hou et al., 2021	South China	30	47	25	5	0	40	7	0
All	China	4173	6137						

Xiao 1, Xiao stage 1; Xiao 2, Xiao stage 2; NA, not publicly available.

### Heterogeneity test and meta-analysis results

**FIGURE 2:**
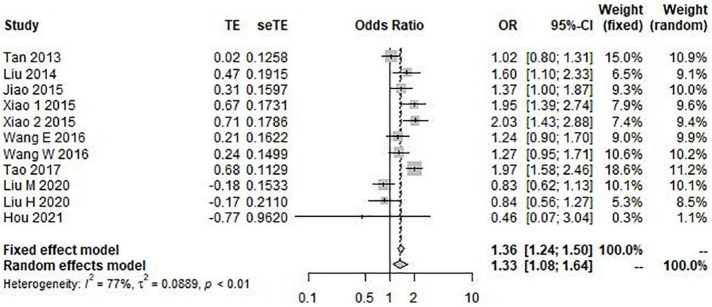
Forest plot for the meta-analysis of the rs9349407 polymorphism using the allele model.

**FIGURE 3:**
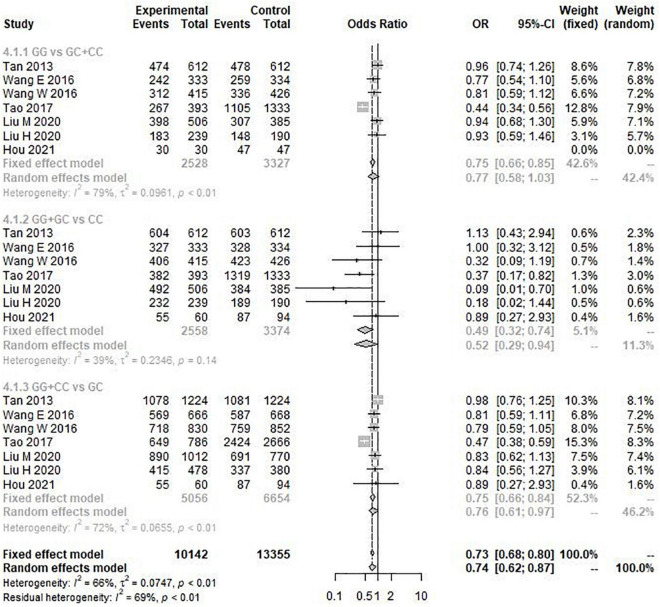
Forest plot for the meta-analysis of the rs9349407 polymorphism using the recessive, dominant, and additive models.

The results of the heterogeneity test showed that allele model (G vs. C) *I*^2^ = 77%, *P* < 0.01, recessive model (GG vs. GC + CC) *I*^2^ = 79%, *P* < 0.01, and additive model (GG + CC vs. GC) *I*^2^ = 72%, *P* < 0.01. Therefore, the three gene models have obvious heterogeneity, so the random effect model is selected for meta-analysis. However, the heterogeneity of the dominant model (GG + GC vs. CC) *I*^2^ = 39% and *P* = 0.14 was not obvious, so the fixed effect model was selected for analysis. Through this analysis, we found that the overall data, including 4,173 cases and 6,137 controls, showed a significant correlation between rs9349407 variation and the genetic risk of AD in allele model (OR = 1.33, 95% CI = 1.08–1.64) (Figure 2), additive model (OR = 0.74, 95% CI = 0.62–0.87), and dominant model (OR = 0.49, 95% CI = 0.32–0.74) (Figure 3). In the recessive model, no association was found between rs9349407 mutation and AD susceptibility in the Chinese population (OR = 0.77, 95% CI = 0.58–1.03) (Figure 3). More detailed results are shown in Figures 2, 3.

### Sensitivity analysis

Since there was a great degree of heterogeneity among the studies on the rs9349407 variation of the CD2AP gene, the stability of the results was determined by the results of sensitivity analysis. We conducted a sensitivity analysis by removing and including studies one by one, and the analysis results are shown in Table 2. In the results, we found that there was almost no change in heterogeneity after being omitted one by one. Therefore, although the heterogeneity of this meta-analysis was large, the results were very stable.

**TABLE 2:** Sensitivity analysis with each study omitted in the meta-analysis.

Study omitted	Heterogeneity test		Meta-analysis		
		
	*I*^2^ (%)	*P*	OR	95% CI	*P*
None	77.00%	<0.01	1.33	[1.08; 1.64]	<0.0001
Tan et al., 2013	75.70%	<0.01	1.43	[1.30; 1.60]	<0.0001
Liu, 2014	78.80%	<0.01	1.35	[1.22; 1.49]	<0.0001
Jiao et al., 2015	79.20%	<0.01	1.37	[1.23; 1.51]	<0.0001
Xiao et al., 2015	76.60%	<0.01	1.32	[1.20; 1.46]	<0.0001
Xiao et al., 2015	76.20%	<0.01	1.32	[1.20; 1.46]	< 0.0001
Wang et al., 2016	79.00%	<0.01	1.38	[1.25; 1.52]	<0.0001
Wang et al., 2016	79.00%	<0.01	1.37	[1.24; 1.52]	<0.0001
Tao et al., 2017	70.10%	<0.01	1.25	[1.13; 1.40]	<0.0001
Liu, 2020	71.60%	<0.01	1.44	[1.30; 1.60]	<0.0001
Liu, 2020	76.10%	<0.01	1.40	[1.27; 1.55]	<0.0001
Hou et al., 2021	78.50%	<0.01	1.37	[1.24; 1.50]	<0.0001

*P*, *P*-values; OR, odds ratio; 95% CI, 95% confidence interval.

### Publication bias analysis

**FIGURE 4:**
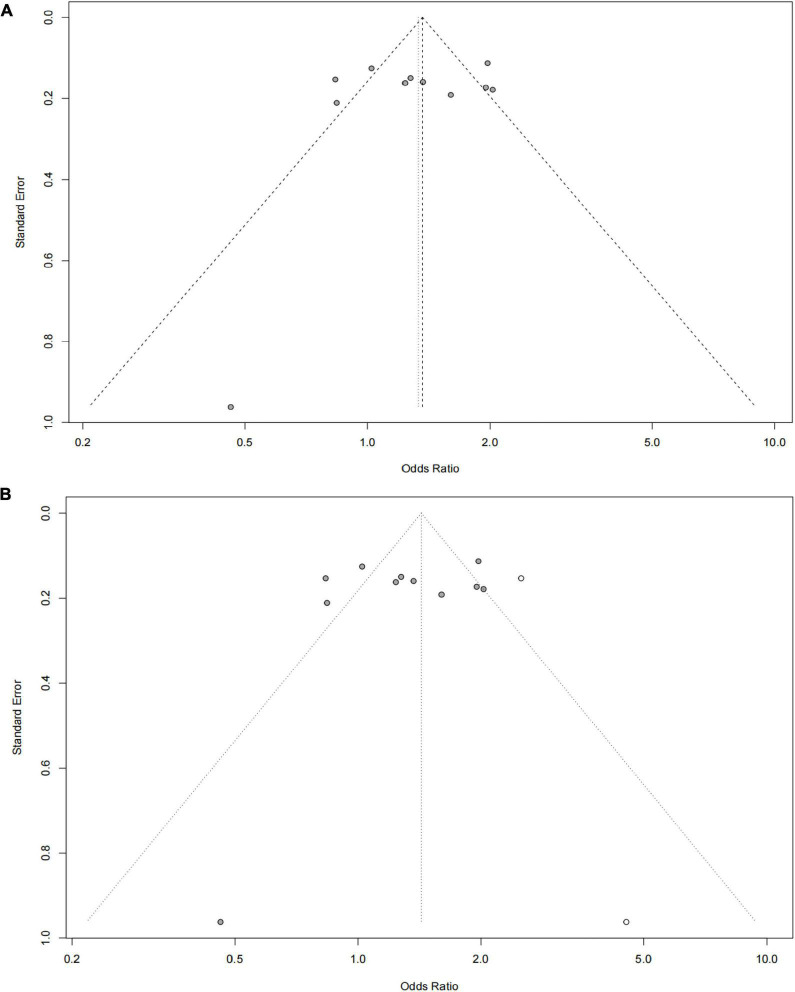
Funnel plot for publication bias analysis of rs9349407 polymorphism in Alzheimer’s disease (AD) using the allele model. **(A)** An inverted funnel plot of selected studies on the relationship between rs9349407 and Alzheimer’s disease. **(B)** A completely symmetric inverted funnel plot of the two studies was filled on the right side of 4A based on the shear complement method.

**FIGURE 5:**
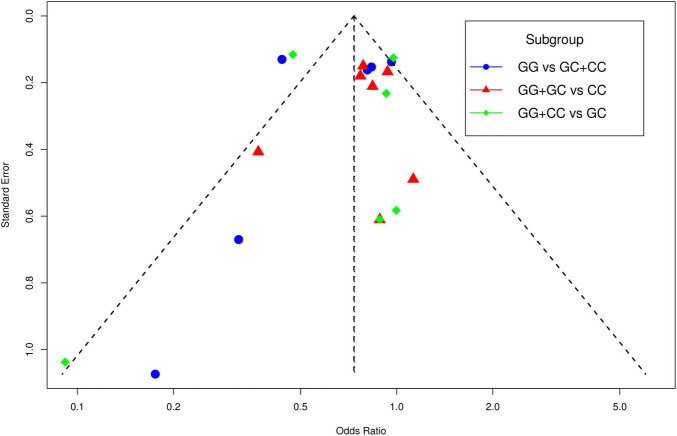
The funnel plots for publication bias analyses of selected studies investigating the association between rs9349407 and Alzheimer’s disease (AD).

The funnel plot of selected studies on the relationship between rs9349407 and AD was an incompletely symmetrical inverted funnel (Figure 4A). According to Begg’s test and Egger regression analysis, the possibility of publication bias in this meta-analysis was revealed (*P* < 1.00E-04). Therefore, the shear complement method was used to evaluate its impact on the meta-analysis results. Two studies were filled in the right side of the funnel plot, which showed a completely symmetric inverted funnel plot (Figure 4B). Subsequently, in the subgroup analysis of additive, dominant and recessive models, it was found that there was no significant publication offset only in the dominant model (GG + CC vs. GC: *P* = 3.00E-04; GG + GC vs. CC: *P* = 1.46E-01; GG vs. GC + CC: *P* < 1.00E-04) (Figure 5). Further analysis of this result, we believe that the main reasons that may lead to publication bias are: (1) Our meta-analysis inadvertently excluded unpublished studies; (2) In the small sample study, more sensitive populations were selected, such as the Mongolian population and the Han population living in Mongolia.

## Discussion

It is reported for the first time from GWAS that the CD2AP rs9349407 polymorphism is significantly associated with AD of Caucasian descent to continuous duplication in the Caucasian population (Logue et al., 2011; Ebbert et al., 2014; Omoumi et al., 2014). All these cases indicate that the link between CD2AP rs9349407 and AD has been well confirmed by GWAS and candidate gene studies in European populations. Meanwhile, the potential role of CD2AP in aging and AD has been extensively investigated. It is reported that CD2-associated protein (CD2AP) is located on chromosome 6q12 and is a protein encoded by the CD2AP gene in humans. In terms of molecular structure, the protein includes three consecutive SH3 domains at the amino terminal, a proline-rich sequence in the intermediate region, and at the carboxyl terminal, a double helix structure area and an actin-cytoskeleton binding site (Cummins et al., 2018). The special molecular structure determines that the protein not only binds and aggregates CD2 to stabilize T cells and antigen-presenting cells but also participates in cytoskeletal (dynamic actin) recombination and intracellular transport (Ghosh et al., 2022).

Although a link between CD2AP deficiency and glomerular pathology has been described, little is known about the function of CD2AP in the brain. The following evidence suggests that the potential pathways of CD2AP in the pathogenesis of AD mainly include four aspects. (1) CD2AP is connected to AD neuroinflammation; CD2AP is expressed in brain neurons and microglia, the central nervous system’s primary immune cells. In AD patients and mouse models, microglia dysfunction has also been identified (Efthymiou and Goate, 2017; Cao and Zheng, 2018; Hickman et al., 2018). (2) CD2AP is associated with the generation of Aβ42; On the one hand, the experimental results of Liao et al. (2015) reported that CD2AP mainly affected Aβ level and Aβ42/Aβ40 ratio, while having no significant effect on Aβ metabolism (Tao et al., 2019). On the other hand, CD2AP also regulates Aβ production in early dendritic endosomes by neuron-specific polarization of Aβ (Ubelmann et al., 2017). (3) CD2AP is connected to tau neurotoxicity. A recent study found that cindr (a human homolog of AD2AP) is a regulator of tau-mediated disease mechanisms. Knockdown of cindr in AD Drosophila has enhanced tau neurotoxicity (Shulman et al., 2014). (4) CD2AP is associated with the complex cellular mechanisms underlying tangles in the brain and tau pathology. In 2021, CD2AP neuronal inclusions similar to NFTs and neuropil thread-like deposits have been in brain from AD patients. Immunofluorescence analysis was performed and showed that CD2AP colocalized with pTau (CD2AP immunoassay was strongly and positively correlated with Braak neurofibrillary stage), whereas in primary tau lesions [corticobasal ganglia degeneration (CBD), progressive supranuclear palsy (PSP), and pick disease (PiD)] cases had no detectable neuronal CD2AP deposits in the samples. These results suggest that CD2AP neuronal expression is associated with 3R-tau-disease (Camacho et al., 2022). Additionally, various studies have suggested that CD2AP is involved in autophagy signaling, the blood-brain barrier, as well as regulating neurite length, neurite complexity, and growth cone filopodia in neurons (Harrison et al., 2016; O’Keefe and Denton, 2018; Sweeney et al., 2018).

Since 2013, the correlation between rs9349407 and AD in the Chinese population has become a research hotspot in recent years. In this paper, we searched five databases, including PubMed, Google Scholar, and CNKI, based on strict inclusion and exclusion criteria and found 11 independent studies with case-control experimental design as the deadline was on 1 April 2022. Although the above studies assessed the potential association between rs9349407 and AD risk in the Chinese population, no consistent conclusion were published. For example, six studies (Tan et al., 2013; Wang et al., 2016; Liu, 2020; Hou et al., 2021) did not find that rs9349407 played an important role in AD susceptibility in the Chinese population. Paradoxically, five studies (Liu, 2014; Jiao et al., 2015; Xiao et al., 2015; Tao et al., 2017) confirmed that rs9349407 polymorphism can increase the risk of AD in Chinese. Based on the inconsistent results reported in recent studies in the Chinese population, we reassessed this association in a relatively large sample (*N* = 10,310; 4,173 AD individuals and 6,137 healthy controls) based on the above 11 studies. In this study, it was revealed that the CD2AP rs9349407 polymorphism in the allele model can increase the susceptibility to AD in the Chinese population, which is consistent with the effect observed in the Caucasian AD cohort. In addition, subgroup analysis showed that rs9349407 variation under the additive model and the dominant model was also significantly correlated with AD susceptibility in the Chinese population (GG + CC vs. GC: *P* = 2.04E-02; GG + GC vs. CC: *P* = 8.51E-04), while no correlation was found between them in the recessive model (GG vs. GC + CC: *P* = 7.44E-02). In conclusion, this updated meta-analysis provides strong evidence for the involvement of the CD2AP rs9349407 variant in AD susceptibility in the Chinese population.

Nevertheless, there are some inevitable limitations to this study, and we hope that future research can avoid them as much as possible. First, the sample size of the included studies is quite limited, which may have varying degrees of impact on the reliability of some subgroup analysis results. Therefore, further large-scale replication studies in the Chinese population are still needed. Second, unpublished studies may be unintentionally excluded from our meta-analysis and more sensitive populations were selected in the small sample studies, which may lead to publication bias. Third, our results are uncorrected estimates and do not consider the influence of confounding factors such as age, gender, environment, education level, and lifestyle habits. Therefore, if there is more detailed personal data, we can conduct a more accurate hierarchical analysis to eliminate the interference of confounding factors and prove our conclusion. The fourth is that AD is a complex disease about the interaction between genes and genes as well as between genes and the environment. However, the studies included in our meta-analysis did not assess the interaction between the rs9349407 variant of the CD2AP gene and other genes or the environment. The last limitation is related to the diagnosis of AD. At present, the diagnosis of AD is mostly based on DSM, CERAD, NINCDS-ADRDA, and other diagnostic criteria, most of which do not provide pathological evidence for further confirmation. Therefore, misdiagnosis and missed diagnosis of AD cannot be ruled out in the included studies. Although this meta-analysis has limitations, it is the most comprehensive meta-analysis to date of the association of the rs9349407 variant of the CD2AP gene with susceptibility to AD in the Chinese population.

In conclusion, this meta-analysis indicates that there is a significant correlation between the rs9349407 variant and AD risk in the Chinese population. However, more detailed genetic studies on AD with a large sample size are still needed to further prove our conclusion in the future.

## Data availability statement

The original contributions presented in this study are included in the article/supplementary material, further inquiries can be directed to the corresponding authors.

## Author contributions

SG and G-YL designed the study. SG, J-WH, Y-NZ, XL, TW, Z-FH, and J-YS analyzed the data. SG, J-WH, Y-NZ, XL, TW, Z-FH, and G-YL wrote the manuscript. J-YS and B-LS put forward constructive modification suggestions and participated in the revision of the article. All authors contributed to the article and approved the submitted version.

## Footnotes

1
https://scholar.google.com/

2
https://cnki.net/

3
https://pubmed.ncbi.nlm.nih.gov/

4
https://www.webofscience.com/wos/author/search

5
https://g.wanfangdata.com.cn/

6
https://www.r-project.org/
